# CSPα in neurodegenerative diseases

**DOI:** 10.3389/fnagi.2022.1043384

**Published:** 2022-11-17

**Authors:** Liqin Huang, Zhaohui Zhang

**Affiliations:** Department of Neurology, Renmin Hospital, Wuhan University, Wuhan, China

**Keywords:** cysteine string proteins α, *DNAJC5*, adult-onset neuronal ceroid lipofuscinosis, neurodegenerative diseases, Alzheimer's disease, Parkinson's disease

## Abstract

Adult-onset neuronal ceroid lipofuscinosis (ANCL) is a rare neurodegenerative disease characterized by epilepsy, cognitive degeneration, and motor disorders caused by mutations in the *DNAJC5* gene. In addition to being associated with ANCL disease, the cysteine string proteins α (CSPα) encoded by the *DNAJC5* gene have been implicated in several neurodegenerative diseases such as Alzheimer's disease (AD), Parkinson's disease (PD), and Huntington's disease. However, the pathogenic mechanism responsible for these neurodegenerative diseases has not yet been elucidated. Therefore, this study examines the functional properties of the CSPα protein and the related mechanisms of neurodegenerative diseases.

## Introduction

Adult-onset neuronal ceroid lipofuscinosis (ANCL), also known as autosomal dominant Kufs disease or Parry disease, is a human neurodegenerative disease characterized by progressive neuronal dysfunction and premature death (Kashyap et al., 2014). The symptoms of ANCL have a wide range of clinical variability, but common symptoms include generalized epilepsy, dyskinesia, and progressive cognitive dysfunction. Seizures are usually the first attack symptom in most patients, and occasionally, other mild features such as behavioral changes, progressive tremors, myoclonus, memory loss, and frequent falls can precede seizures (Naseri et al., 2021). Abnormal electroencephalogram (EEG) readings were found in all reported patients during their initial clinical manifestations. A recent experience demonstrated that the EEG abnormalities at earlier stages of the disease showed a predominance of diffuse theta and slow delta waves during wakefulness, intermittent delta activity, and generalized spike-wave and polyspike-wave complexes, and these changes become more prominent during disease progression (Naseri et al., 2021). Patients with the onset of the seizures show a progressive cognitive decline, ataxia, memory loss, and speech impairment. Some patients may show signs of depression (Naseri et al., 2021). Patients with ANCL typically die between the ages of 30 and 60; however, this seems to vary with the onset of the seizures. The causes of death are severe neurological damage and multiple organ failure (Naseri et al., 2021).

Human genetic studies have successfully identified single gene mutations in various neurodegenerative diseases, such as frontotemporal dementia (FTD), Parkinson's disease (PD), and Huntington's disease, which are associated with alterations in genes encoding Tau, α-synuclein, and protein Huntingtin, respectively (Ross and Poirier, 2004). Misfolding and aggregation of these and other proteins have been implicated in disease progression, and it is thought that defective protein homeostasis is a common underlying feature of neurodegeneration (Calamini and Morimoto, 2012). ANCL is no exception, and it was found to be caused by mutations in a gene called *DNAJC5* (Nosková et al., 2011; Cadieux-Dion et al., 2013; Jarrett et al., 2018). There are three subtypes of the *DNAJC5* gene, *DNAJC5a, DNAJC5b*, and *DNAJC5g*, which encode three different cysteine string proteins (CSP) α, β, and γ, respectively. CSPα is mainly expressed in neuronal cells and other secretory cells (Fernández-Chacón et al., 2004), and it is also the main pathogenic protein in ANCL pathogenesis (Nosková et al., 2011). Nevertheless, the expression patterns of CSPβ and CSPγ are controversial (Eybalin et al., 2002), and their expression and function appear to be limited to the testis (Gorleku and Chamberlain, 2010).

## CSPα

### Structure of CSPα

CSPα protein is known as a cysteine string protein because it has a motif containing 12 to 14 cysteine residues (Chamberlain and Burgoyne, 2000). CSPα is an evolutionarily conserved member of the DnaJ/heat shock protein 40 (HSP40) family (Haltia, 2003). HSP40, the largest class of HSPs, is involved in the assembly and disassembly of macromolecular complexes (Roosen et al., 2019). Another name for the HSP40 family is the J-domain protein (JDP) family, which is an essential co-partner of HSP70 (Kampinga et al., 2019). As the name implies, a J-domain in JDP plays a key role in stimulating the ATPase activity required for HSP70 to utilize the substrate conformation (Kampinga and Craig, 2010). JDPs deliver misfolded protein substrates to HSP70 and activate HSP70-ATPase *via* the highly conserved histidine-proline-aspartate (His-Pro-Asp) motif in the J-domain between helices II and III (Kampinga and Craig, 2010; Lee et al., 2022a). The process plays an important role in protein translation, folding, unfolding, translocation, and degradation (Qiu et al., 2006). Expression of JDPs is typically restricted to specific tissues and cell types or subcellular compartments, conferring specificity by recruiting HSP70 to defined protein complexes. JDPs are responsible for the functional diversity of HSP70 proteins (Hageman and Kampinga, 2009). The human genome encodes 41 JDPs, and CSPα is a member of the JDP family with a unique cysteine residue string (Kampinga and Craig, 2010). CSPα has five distinct domains: a small N-terminal segment, a J-domain homologous to the bacterial ancestor protein DnaJ, consisting of four tightly folded α-helices (Lee et al., 2022a), a central cysteine string domain (CSD) with 12 to 14 cysteine residues, a hydrophobic linker, and a vaguely named C-terminal domain (Naseri et al., 2021). The central CSD of CSPα is palmitoylated by thioester bonds with cysteine side chains, and this modification enables CSPα to target lipid membranes such as synaptic vesicles and act as a chaperone to control vesicle exocytosis mechanisms, particularly at synapses (Jarrett et al., 2018; Naseri et al., 2021). Thus, in addition to being important for protein homeostasis, CSPα, a highly conserved presynaptic JDP, is essential for synapses to remain operational for long periods of time, although CSPα is not required for synaptogenesis (Fernández-Chacón et al., 2004; Johnson et al., 2010).

### Distribution and function of CSPα

CSPα is widely expressed in a variety of human tissues. In neurons, CSPα is mainly positioned on synaptic vesicles in the presynaptic terminal (Ohyama et al., 2007), and a small portion was detected on lysosomes (Benitez and Sands, 2017). Moreover, individual research has discovered that CSPα can also be detected in cell membranes and melanosomes (Huber, 2016). CSPα, as a special synaptic molecular chaperone in neurons, forms a chaperone complex at the pre-synapse with its binding partners−70 kDa heat-shock cognate protein (HSC70) and small glutamine-rich tetrapeptide repeat protein (SGT)—to accompany the synaptic soluble N-ethyl maleimide sensitive factor attachment protein receptor (SNARE) protein synaptosome associated protein of 25 kDa (SNAP-25) and maintain its conformation (Kashyap et al., 2014; Jarrett et al., 2018), facilitating the formation of synaptic SNARE complexes (including SNAP-25, VAMP2 and Syntaxin-1), which are required for the fusion of synaptic vesicles with the plasma membranes (Kashyap et al., 2014; Jarrett et al., 2018; Jedlickova et al., 2020; Naseri et al., 2021). CSPα can also interact with the chaperone dynamin to control synaptic protein folding and trafficking (Sharma et al., 2012b) and consequently regulate a variety of cellular processes, including calcium homeostasis (Bronk et al., 2005), membrane fusion (Bronk et al., 2001), neurotransmitter release (Wu et al., 1999), and synaptic stability (Donnelier and Braun, 2014). In non-neuronal cells, CSPα was prominently localized to late endosomes/lysosomes, with some detected in the perinuclear compartment and some at the cell surface (Xu et al., 2018; Lee et al., 2022b). Indeed, CSPα is not a membrane protein, but it contains a string of cysteine residues that can be palmitoylated (Burgoyne and Morgan, 2015). Therefore, CSPα can anchor to the membranes of late endosomes and lysosomes by lipid modification (Ye, 2018). CSPα in non-neuronal cells is mainly associated with endolysosomes, but the function of endolysosome-associated CSPα is still unclear (Benitez and Sands, 2017). Furthermore, CSPα also appears to have functions outside the nervous system. Early published research, for example, underlined that CSPα is associated with the secretion of insulin by pancreatic β-cells (Brown et al., 1998), and CSPα immunoreactivity was observed to be lower in type 2 diabetic Goto-Katizaki rats in another study, but the specific mechanism remains unclear (Johnson et al., 2010).

## CSPα and ANCL

### CSPα mutations and pathological changes in ANCL

**Figure 1:**
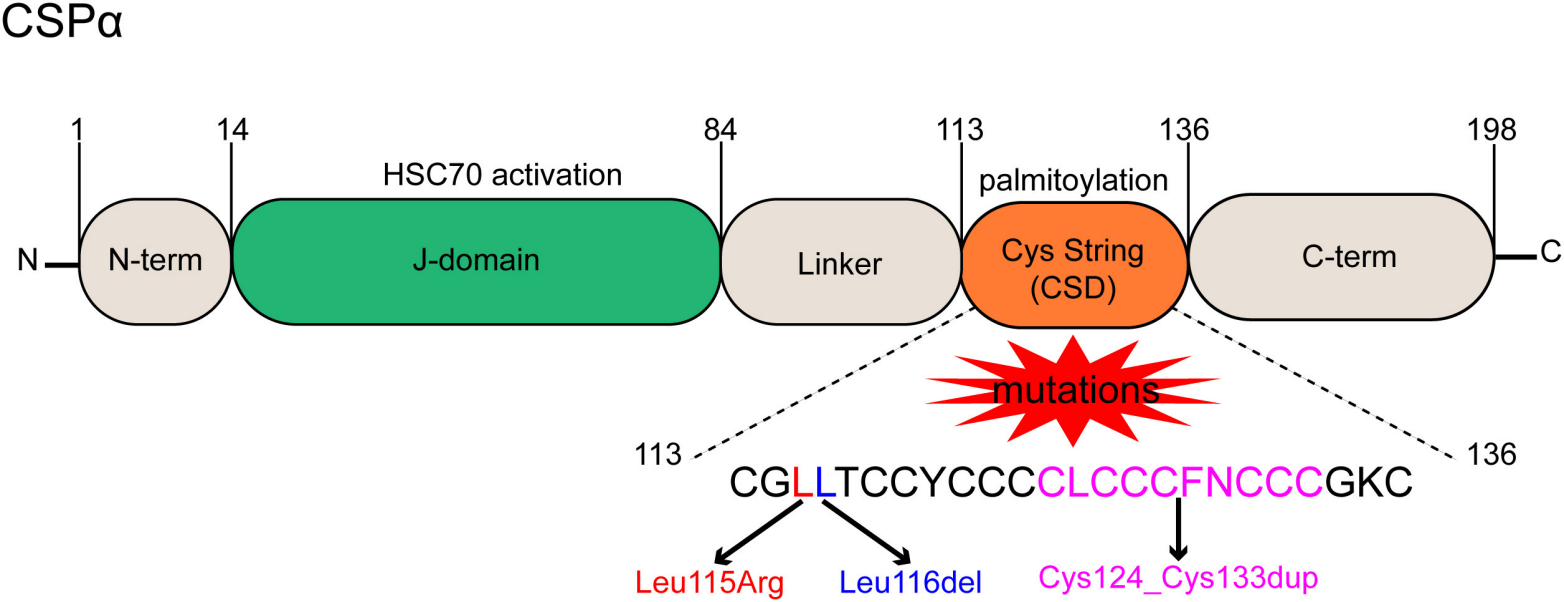
Functional domains and mutant forms of CSPα. CSPα consists of 5 domains, a small N-term domain, a J-domain is essential for HSC70 interaction and activation, a hydrophobic linker, a cysteine string domain (CSD) containing cysteine residues for palmitoylation, and a disordered C-term domain. Three mutations result in ANCL, a single base change replaces Leu115 with an arginine residue (p.L115R), a loss of Leu116 caused by deletion of three nucleotides (p.L116δ), and a 30-bp intra-frame duplication (Cys124_Cys133dup) of the central core motif in the CSD.

Two mutations in *DNAJC5* are related to the occurrence and progression of ANCL (Yao et al., 2017). One is a single base change that replaces Leu115 with an arginine residue (p.L115R), and the other is the loss of Leu116 caused by the deletion of three nucleotides (p.L116δ) (Diez-Ardanuy et al., 2017). Besides, a new mutation of *DNAJC5* that can also lead to ANCL was reported in a newly published study. It is a 30-bp intra-frame duplication of the central core motif in the CSD of *DNAJC5* (Cys124_Cys133dup), and it reportedly affects palmitoylation-dependent CSPα localization in cultured neuronal cells (Jedlickova et al., 2020) (Figure 1).

On the level of the histopathological change, ANCL, as with all neuronal ceroid lipofuscinoses (NCLs), is characterized by a large amount of auto-fluorescent lysosomal storage material (AFSM), which is also known as lipofuscin (Sadzot et al., 2000), and it also showed neurodegeneration in neurons in the cerebral cortex, striatum, amygdala, hippocampus, substantia nigra, and cerebellum (Kashyap et al., 2014; Naseri et al., 2021). Lipofuscin bodies contain two major proteins, saposin and the C-subunit of mitochondrial ATPase (Naseri et al., 2021). However, a past study has shown that the cerebral lysosomal inclusions of Patients with ANCL are rich in saposin-D but not the C-subunit of ATPase (Nijssen et al., 2003), indicating that the composition of lipofuscin is still controversial and more research is needed to verify it. In addition, lipid analysis has identified free fatty acids such as palmitic and arachidonic acids as the major lipid components in lipofuscin, possibly due to increased phospholipase activity and/or abnormal membrane transport (Bazan et al., 1990).

Neurodegeneration in neurons is a prominent pathological feature of ANCL. It was reported that neuronal cell death appears to be related to age at onset and disease duration in most NCLs. It is usually most pronounced in infantile NCL, while in juvenile NCL, it may be moderate, and in adult NCL, it may be rather mild (Williams et al., 2006). Unfortunately, nearly all of the understanding of ANCL is based on the analysis of postmortem tissue from terminal cases (Nosková et al., 2011). Similar findings are reported in at least ten published neuropathology studies of patients with advanced ANCL: macroscopically, symmetric cerebral and cerebellar atrophy and depigmentation of the substantia nigra are consistently reported. Light microscopy shows significant neuronal depletion, extensive synaptic degeneration in their frontal, parietal, and temporal cortex, and consistent accumulations of AFSM (Ferrer et al., 1980; Josephson et al., 2001; Burneo et al., 2003; Nijssen et al., 2003). In recent years, research has described the pathological features of a patient in a clinically early stage of ANCL with a *DNAJC5* p.L115R mutation. The pathology of this patient shows typical intracellular ceroid accumulation in neurons and early neuroinflammation but no evidence of cerebral atrophy, neurodegeneration, or massive synaptic loss. Similarly, there is little or no evidence of neuronal loss in the examined areas (Benitez et al., 2015). Besides, microgliosis and astrogliosis appear to be early events that may even precede neuronal degeneration in ANCL and remain significant in late neurodegeneration in the cerebral cortex (Williams et al., 2006; Benitez et al., 2015). Another fascinating discovery in research by Benitez et al. was that compared to the terminal cases in which the postmortem brain samples displayed a large reduction in CSPα levels (Nosková et al., 2011; Greaves et al., 2012; Benitez et al., 2015; Donnelier et al., 2015), there were no significant differences in CSPα or synaptophysin in the neuropil in the patient in an early stage of ANCL (Benitez et al., 2015). Thus, although the effect of these mutations on CSPα and how this results in ANCL have not been established, the above findings seemingly confirm a neuroprotective role for CSPα in humans and demonstrate that the resulting depletion of CSPα might cause in parallel the presynaptic dysfunction and the progressive neurodegeneration observed in affected individuals and lysosomal accumulation of misfolded and proteolysis-resistant proteins in the form of characteristic ceroid deposits in neurons (Burgoyne and Morgan, 2015). Besides, given that CSPα mainly concerns with the function of synapses and lysosomes, as noted earlier, the extensive neurodegeneration seen in patients with advanced ANCL appears to be the result of the early effects (most likely on lysosomal function) of mutated CSPα in the body and subsequent neuronal loss and synaptic dysfunction due to reduced CSPα levels. Lysosomal dysfunction (ceroid accumulation) occurs before neuronal cell death and synaptic degeneration (Burgoyne and Morgan, 2015).

### Changes in protein levels

The alteration in protein levels of CSPα in patients with ANCL has been known through the introduction above. Realistically, a variety of additional proteins demonstrate altered expression patterns in ANCL. For instance, Henderson and collaborators (Henderson et al., 2016) probed the changes in protein levels in the brains of patients with ANCL using either a *DNAJC5* p.L115R or a *DNAJC5* p.L116δ mutation. They found that 14 protein levels were upregulated, and compared to matched controls; three protein levels were downregulated in p.L115R and p.L116δ ANCL brains. The most significant changes were in palmitoyl-protein thioesterase 1 (PPT1) and saposin-D, which increased by about 21-fold. As discussed, saposin-D is a protein enriched in lysosomal inclusions of Patients with ANCL. Thus, its elevated levels are consistent with the composition analysis based on lipofuscin bodies (Nijssen et al., 2003). PPT1, the other protein with a significantly high level, removes acyl chains from palmitoylated proteins during degradation. Although PPT1 was originally thought to be a lysosomal enzyme in non-neuronal cells, it has now been shown that PPT1 localizes to axons and presynaptic terminals in neurons (Kim et al., 2008). Intriguingly, PPT1, like CSPα, has been demonstrated to be associated with another NCL disease, ceroid lipofuscinosis neuronal 1 (CLN1), which is one of the two infantile forms of NCL and the most severe subtype of the disease (Cárcel-Trullols et al., 2015). Furthermore, PPT1 has been reported to play a key role in regulating the synaptic vesicle cycle, and PPT1 knockout (KO) neurons exhibit synaptic vesicle cycle defects (Aby et al., 2013). Notably, in addition to increasing PPT1 levels, CSPα mutations also affect PPT1 expression, localization, and enzymatic activity, resulting in changes in the palmitoylation of global proteins, especially lysosomal and synaptic proteins (Henderson et al., 2016). These observations have unambiguously established a link between palmitoylation and ANCL (Diez-Ardanuy et al., 2017) and a strong functional association between NCL-related genes (Henderson et al., 2016).

As for the precise change of CSPα levels, Henderson and collaborators found that 0.66-fold significantly reduced CSPα levels in patients ANCL by using either a *DNAJC5* p.L115R or a *DNAJC5* p.L116δ mutation (Henderson et al., 2016), which is in line with other research that measured CSPα levels in brain samples from an advanced ANCL patient with a *DNAJC5* p.L116δ mutation and found a 50% reduction (Donnelier et al., 2015), indicating that ANCL occurring in humans is the result of reduced levels of CSPα. Notably, Henderson and collaborators measured membrane-bound CSPα levels in brain samples from advanced Patients with ANCL with a *DNAJC5* p.L115R mutation and found a 50% reduction (Benitez et al., 2015). Indeed, CSPα is a cytosolic and membrane-bound co-chaperone (Zinsmaier, 2010), and CSPα has reportedly exhibited chaperone and co-chaperone activity independent of its ability to bind membranes (Benitez et al., 2015), which seems to indicate that measuring membrane-bound CSPα is of little significance for ANCL. More attention should be paid to cytosolic CSPα. Nevertheless, we cannot rule out the possibility that the overall reduction, up to a certain ratio of the control levels, in both soluble and membrane-bound CSPα found in Patients with ANCL could be responsible for the synaptic defects.

In addition to the proteins mentioned above, whose levels vary significantly, are there any changes in the levels of other proteins whose functions are closely related to CSPα, such as SNAP-25 and HSC70? The answer is yes. Several lines of evidence suggest that depletion of CSPα interferes with SNARE complex formation and profoundly affects presynaptic vesicle release and synaptic function (Fernández-Chacón et al., 2004; Burgoyne and Morgan, 2011; Sharma et al., 2011). Indeed, the SNARE complexes forming presynaptic proteins, SNAP-25, were remarkably reduced in Patients with ANCL (Sharma et al., 2011; Zhang et al., 2012; Benitez et al., 2015; Donnelier et al., 2015). Notably, the sample identified as clinically suspected ANCL but negative for mutations in *DNAJC5* did not have a similar reduction in CSPα at either the mRNA or protein levels (Donnelier et al., 2015), underscoring that deficiency of SNAP-25 and hence reduced SNARE complex formation is thought to be the downstream consequence of impaired CSPα function (Kashyap et al., 2014). Interestingly enough, HSC70, as a crucial chaperone of CSPα, lacks variation in patients with ANCL compared to normal control individuals (Benitez et al., 2015; Henderson et al., 2016; Benitez and Sands, 2017) and shows a tendency to decrease (Henderson et al., 2016). On the face of it, this result seems bizarre. However, given that HSC70 is widely expressed in many tissues and evolutionarily conserved in all eukaryotes, even bacteria, while CSPα is mainly expressed in the brain and tissues specialized for exocytosis, it is absent in lower eukaryotes such as yeast. These differences in tissue expression patterns and evolution imply that HSC70 performs functions independent of CSPα, possibly by collaborating with other J-domain proteins. The result makes sense (Tobaben et al., 2001). Indeed, the function of HSC70 has been proven to be independent of CSPα, and HSC70 has been shown to interact with several different J-domain and tetratricopeptide repeat (TPR) domain proteins (Tobaben et al., 2001).

Besides, ANCL-related mutations have been reported to reduce CSPα palmitoylation (Naseri et al., 2020). These mutants are also more prone to aggregation (Diez-Ardanuy et al., 2017; Imler et al., 2019), seemingly consistent with the significant increase in PPT1 levels mentioned above. In practice, palmitoylation has long been linked to neurodegeneration (Boehme et al., 1971). Nevertheless, the pathogenesis of palmitoylation leading to ANCL remains unclear. Another protein involved in palmitoylation modification is HIP14/DHHC17, a protein acyltransferase that has been considered responsible for CSPα aggregation in DHHC palmitoyl-dependent ANCL (Zhang et al., 2012). However, the detection of HIP14/DHHC17 showed that the levels of this enzyme were unchanged in brain samples of patients with ANCL (Henderson et al., 2016). Comparatively speaking, ANCL-related palmitoylation appears to be more closely related to PPT1. Increased levels and decreased activity of PPT1 caused by ANCL-dependent CSPα mutations have been reported to affect lysosomal function. Lysosomal dysfunction has also been seen as the basis of NCLs, whereupon research by Henderson et al. detected other lysosomal enzyme-associated proteins, including TPP1/CLN2, ATP6V1A, and LC3B II; they found no change in these proteins in the brains of patients with ANCL (Henderson et al., 2016). Although the mechanism of lysosomal dysfunction is unclear, these results indicate that lysosomal dysfunction in ANCL is primarily mediated through PPT1.

It is remarkable. However, the changes in protein levels or phenotype caused by CSPα mutations or deletions in animals do not appear identical to those in humans. Research with *Dnajc5* gene KO mice has shown that this mutation can cause neurodegeneration. However, *Dnajc5* hemizygous mice with a 50% reduction in CSPα levels showed a normal phenotype (Fernández-Chacón et al., 2004), suggesting that other, as-yet-unidentified mechanisms contribute to the neurodegeneration beyond the reduction of CSPα levels. One potential explanation offered by the research is that the relatively mild phenotype of mouse *Dnajc5* mutants may be due to the compensatory expression of CSPβ, which is highly homologous to CSPα (Gundersen et al., 2010). However, the hypothesis remains highly controversial as CSPβ, and CSPγ mRNA were reportedly detected only in testis (Fernández-Chacón et al., 2004). Possible mechanisms should be further discussed.

Moreover, according to a report, CSPα protein deletion did not affect PPT1 expression in CSPα KO mice brains, except for a slight increase in its activity, underscoring the fact that the change in PPT1 levels is not caused by CSPα deletion but by changes in palmitoylation modifications resulting from CSD domain mutations of CSPα (Henderson et al., 2016). Consistent with ANCL presentation in humans, SNAP-25 protein levels and synaptic SNARE complex assembly were reduced by ~50% in the brains and primary neurons of CSPα KO mice (Sharma et al., 2012b; Naseri et al., 2021). Indeed, a detailed analysis of CSPα KO mice suggests that neurodegeneration in CSPα-deficient mice results from functional defects in SNAP-25 and SNARE binding proteins (Sharma et al., 2012b). Unfortunately, the lysosomal function has not been evaluated in CSPα-deficient mice (Donnelier and Braun, 2014; Naseri et al., 2021).

### CSPα palmitoylated and aggregation

CSPα has the highest palmitoylation level of any known brain protein. As mentioned above, mutant CSPα has reduced palmitoylation and is more prone to aggregation, which is consistent with the finding that high-molecular-weight anti-SDS resistant aggregates formed by CSPα exist in postmortem brain tissues of patients (Diez-Ardanuy et al., 2017). CSPα aggregation is thought to trigger ANCL neurodegeneration (Diez-Ardanuy et al., 2017).

We further investigated the palmitoylation of CSPα mutants and the mechanisms that mediate CSPα aggregation in ANCL. Past research revealed that wild-type (WT) CSPα forms dimers in cells, whereas non-palmitoylated CSPα produced by bacteria (WT CSPα proteins were overexpressed in E. coli) forms higher molecular weight oligomers (Swayne et al., 2003), underscoring the intrinsic propensity for protein self-association and suggesting that a central core of CSPα palmitoyl cysteine is essential to preventing aggregation of CSPα mutants (Diez-Ardanuy et al., 2017). *DNAJC5* p.L115R and *DNAJC5* p.L116δ mutations alter the structure of CSD and disrupt CSPα palmitoylation and membrane targeting by affecting specific dileucine residues within the cysteine string region (Henderson et al., 2016; Benitez and Sands, 2017), resulting in hydrophobic palmitoyl chains in the CSD core that no longer undergo optimal membrane interactions but aggregate through hydrophobic interactions (Diez-Ardanuy et al., 2017; Imler et al., 2019; Naseri et al., 2020; Lee et al., 2022b). These studies emphasize that the central core of palmitoyl cysteine is essential for the aggregation of ANCL CSPα mutants.

Interestingly, compared with CLN1 caused by PPT1 mutation, the proportion of CSPα aggregates in ANCL was very small (Henderson et al., 2016). Given that the levels of the PPT1 in the brain increased 21-fold in patients with CSPα mutations, and PPT1 mutation resulted in large aggregation of CSPα, further discussion into the relationship between PPT1 and CSPα is needed. Henderson and collaborators (Henderson et al., 2016) established *in vitro* depalmitoylation experiments using immunoprecipitated CSPα and recombinant expressed human PPT1, and the results showed that CSPα could be stably depalmitoylated by PPT1, indicating a substrate-enzyme relationship between CSPα and PPT1. Moreover, this research also found that PPT1 can be expected to depalmitoylate CSPα, which may promote its degradation (Henderson et al., 2016). Consistent with this view, a recent study has found that the palmitoylated monomer of the ANCL CSPα mutant has a shorter lifetime than WT CSPα and has speculated that the mutant can be rapidly depalmitoylated or consumed in a time-dependent manner into a high molecular weight polymeric form (Diez-Ardanuy et al., 2017). Then, how to explain why increased PPT1 levels in ANCL lead to decreased palmitoylation of CSPα but do not simultaneously increase the proportion of CSPα aggregation? A likely explanation is that ANCL CSPα mutant aggregation does not depend on palmitoylation but on the intrinsic properties of the mutant itself (Zhang and Chandra, 2014) and that the gain function in ANCL is mainly due to PPT1 accumulation and mislocalization rather than CSPα aggregation (Henderson et al., 2016).

### CSPα and ANCL: Possible signaling pathways

CSPα appears to be associated with molecular mechanisms that regulate or interfere with the proliferation and differentiation of neural stem cells (Nieto-Gonzalez et al., 2019). Radial glioid (RGL) stem cells are a relatively quiescent cell population in the dentate gyrus that undergoes a self-renewal process and commits to an astrocytic or neuronal cell fate (Bonaguidi et al., 2011; Encinas et al., 2011). The key regulatory points for postnatal neurogenesis are the maintenance of quiescence (Song et al., 2012) and neuronal survival (Kempermann et al., 2006). Nieto-Gonzalez and collaborators (Nieto-Gonzalez et al., 2019) found that mouse RGL neural stem cells expressed synaptic cooperative chaperone CSPα. Interestingly, in CSPα KO mice, RGL stem cells lost their quiescence after birth and entered a hyperproliferative state, which increased the production of neural intermediate progenitor cells, thus depleting the hippocampal neural stem cell pool. This finding highlights the importance of molecular chaperones for controlling factors that regulate neural stem cell quiescence (Furutachi et al., 2013) and promoting the survival of nascent neurons (Ramírez-Rodríguez et al., 2013). In particular, on a biochemical level, excessive cell proliferation is caused by excessive activation of the rapamycin (mTOR) signaling pathway, implying a direct or indirect interaction between CSPα/mTOR signaling, which may underlie the molecular mechanism of brain dysfunction and neurodegeneration (Nieto-Gonzalez et al., 2019). Nieto-Gonzalez and collaborators explored proteins and pathways involved in neural stem cell proliferation, including p27 (Andreu et al., 2015), Wnt/β-catenin (Lie et al., 2005), Notch (Imayoshi et al., 2010), sonic hedgehog (Lai et al., 2003), and PI3K/Akt/mTOR (Aberg et al., 2003). They only found changes in the mTOR signaling pathway (Nieto-Gonzalez et al., 2019). They found that in the absence of CSPα, the overactivation of the mTOR signaling pathway was the leading cause of neurogenesis abnormalities, and SK6 kinase activity, a downstream target of mTORC1, was also increased, which may be triggered by the overactivation of mTORC1 (Nieto-Gonzalez et al., 2019). An abbreviated explanation from the researchers suggests that CSPα may play a regulatory role in a key biochemical reaction that maintains quiescence under basal conditions within the IP3K/Akt/mTORC1 cascade (Nieto-Gonzalez et al., 2019). However, the study seems to lack detection of molecules upstream of mTORC1, such as IP3K and Akt. Therefore, the influence of CSPα mutation on the PI3K/AKT/mTOR pathway remains to be worked out. One thing is for certain. Nevertheless, there is a clear connection between CSPα and mTOR.

The mTOR pathway has been extensively implicated in neurodegenerative diseases, mainly because the overactivation of mTORC1 effectively inhibits autophagy (Menzies et al., 2015), and it has been proven that the failure of autophagy degradation is a major culprit in neurodegenerative diseases (Wong and Cuervo, 2010). Although the mechanism by which mutations in CSPα lead to neuronal ceroid lipofuscinosis is not fully elucidated (Nosková et al., 2011), lysosomal dysfunction, abnormal endoplasmic reticulum-lysosomal transport, and aberrant lipid modifications are thought to underlie this disease (Cotman et al., 2013). CSPα correlates with lysosomal dynamics, depending on nutrient levels and mTORC1 activation status (Wyant et al., 2018). TFEB, the master autophagy, and lysosomal biogenesis regulator, has been used as a therapeutic tool in cellular and mouse models characterized by the accumulation of toxic aggregates, including lysosomal storage disorders and neurodegenerative diseases. Furthermore, studies have suggested that the primary mechanism for the subcellular localization of TFEB is regulated by mTOR-mediated phosphorylation (Napolitano et al., 2018). Coincidentally, PPT1 mRNA levels were increased in patients with ANCL, suggesting that the significant increase in this protein was induced by transcriptional genesis, and TFEB was considered the most likely transcription factor involved (Henderson et al., 2016). This study assumes that mutant CSP is more likely to oligomerize (Greaves et al., 2012; Zhang and Chandra, 2014), which in turn impairs synaptic trafficking (Greaves et al., 2012). This, in turn, leads to the transcription of PPT1 *via* the transcription factor TFEB (Sardiello et al., 2009), which ultimately depalmitoylates and degrades mutant CSPα (Henderson et al., 2016). Based on these observations, it is reasonable to hypothesize that there may be a CSPα/mTOR/TFEB/PPT1 signaling pathway in the pathogenesis of ANCL. The pathway leads to neurodegenerative diseases through various cascading reactions. Furthermore, a lack of change in PPT1 levels observed in the study by Henderson et al. following immunoblotting of human cortical samples from AD and various lysosomal storage diseases suggests that increased PPT1 levels not only reflect increased lipofuscin accumulation but may also be selective for ANCL (Henderson et al., 2016).

## CSPα and other neurodegenerative diseases

CSPα has also been linked to several other neurodegenerative diseases (Tiwari et al., 2015; Henderson et al., 2016), including transmissible spongiform encephalopathy, AD, Huntington's disease, PD, amyotrophic lateral sclerosis (ALS), spinocerebellar ataxia type 1 and FTD (Johnson et al., 2010). Published studies have underlined that the HSC70/DnaJ complex is essential for regulating many aspects of neurodegenerative proteins, including their disaggregation, stabilization, degradation, and secretion (Fontaine et al., 2016). For instance, TDP-43, α-synuclein, and microtubule-associated protein Tau could all be driven out of the cell by CSPα (Fontaine et al., 2016; Lee et al., 2018). Based on the discussion above, it can be found that CSPα is also closely associated with other neurodegenerative diseases in addition to ANCL.

### CSPα and AD

CSPα expression was found to be downregulated in the forebrain in the early stage of AD (Mole and Cotman, 2015), and this alteration preceded the decrease of synaptic vesicle protein (Lee et al., 2022b). Presynaptic degeneration is typically known to be key to AD's pathogenesis (Fontaine et al., 2016). Coincidentally, mouse gene knockdown studies have shown that CSPα is essential for synaptic survival (Greaves et al., 2008; Lee et al., 2022b). The downregulation of CSPα expression observed in the AD hippocampus has been suggested to be closely related to early synaptic degeneration and the resulting impaired memory formation in AD (Tiwari et al., 2015). CSPα is thought to have a variety of presynaptic functions, including that (1) CSPα forms the CSPα/SGT/HSC70 chaperone complex with SGT and HSC70, which localizes in synaptic vesicles and interacts with SNARE proteins, leading to calcium-triggered exocytosis of synaptic vesicles (Evans and Morgan, 2002). (2) CSPα regulates presynaptic calcium levels by modulating the activity of presynaptic calcium channels (Ranjan et al., 1998). (3) CSPα regulates endocytosis by promoting dynamin1 polymerization (Tiwari et al., 2015). More precisely, dynamin is an important protein in a clathrin-coated vesicle (CCV) fission. The interaction between CSPα and dynamin can promote dynamin polymerization and thus contribute to intracellular vesicle fission (Olgiati et al., 2014). Furthermore, it has been described that defects in intracellular fission may lead to a decrease in the number of synaptic vesicles prone to exocytosis (Krebs et al., 2013). Together, these findings indicate that CSPα is also important for normal synaptic function, and defects in the extracellular machinery of CSPα KO mice may be due to defects in the CSPα-dependent endocytosis machinery (Roosen et al., 2019). (4) CSPα regulates the density of calcium-dependent K^+^ (BK) channels in the presynaptic terminal, keeping excitability in check. Previous investigations have found that presynaptic BK channel activation can reduce basal synaptic transmission in the hippocampal CA1 region of the mouse AD model while reducing CSPα expression is expected to increase presynaptic BK channel density at synapses and thereby reduce presynaptic terminal excitability (Tiwari et al., 2015). The previously mentioned synapse-related functions of presynaptic vesicle protein CSPα imply that it may play a key role in synaptic degeneration and protection in AD (Tiwari et al., 2015).

### CSPα and PD

Gene mutation or gene duplication of α-synuclein encoding gene *SCNA* is widely associated with familial PD (Stefanis, 2012). Aggregation of α-synuclein at synapses is considered an early PD event associated with impaired striatal synaptic function and dopaminergic neuron death (Caló et al., 2021). PD-related protein α-synuclein was found to assist CSPα in the correct assembly of SNARE complexes (Roosen et al., 2019), indicating that α-synuclein may be a regulator of synaptic exocytosis or vesicle cycling. Notably, the increased expression of α-synuclein can rescue the degeneration of presynaptic nerve endings in CSPα-deficient mice (Johnson et al., 2010) but does not rescue the downregulation phenotype of SNAP-25, indicating that α-synuclein is crucial for the integrity of synaptic nerve endings (Johnson et al., 2010), as well as α-synuclein may be downstream or parallel to SNAP-25 in membrane transport (Lee et al., 2022a). Additionally, α-synuclein aggregating at the synapse is related to a reduction of synaptic CSPα and a decrease of the complex formed by CSPα with HSC70 and STG.

Conversely, an increase in CSPα can reduce α-synuclein aggregation and increase monomeric a-syn while rescuing striatal dopamine release impairment (Caló et al., 2021). Despite this, it is unclear how α-synuclein erases neurodegeneration triggered by CSPα deletion. What is certain is that CSPα and relevant chaperones could be identified as therapeutic tools to restore normal synaptic function in early-stage PD.

### CSPα and Huntington's disease

Huntington's disease is also reported to be a dominant neurodegenerative disorder associated with abnormal palmitoylation and caused by alterations in HIP14/DHHC17 (Henderson et al., 2016). It has long been known that HIP14/DHHC17 can palmitoylate CSPα and its substrate. Interestingly, when the palmitoylation of CSPα is blocked, the formation of inclusions containing mutant huntingtin increases (Diez-Ardanuy et al., 2017). CSPα, in reality, has been shown to interact with Huntington proteins (Jarrett et al., 2018). Johnson and collaborators (Johnson et al., 2010) found that CSPα binds to polyglutamine-amplified mutant Huntington protein but not to WT Huntington protein in transiently transfected cells, suggesting that neurodegeneration in Huntington's disease is associated with CSPα sequestration. Given the long-term higher toxicity levels and/or expansion of Huntington's in patients, CSPα will eventually be depleted, and its lack of available neuroprotection will be a prominent mechanism of Huntington's disease.

## CSPα and protein quality control

It has been reported that the most likely cause of the neurodegenerative diseases induced by CSPα defects is the progressive misfolding of one or more client proteins and dysregulation of unconventional protein secretion (Johnson et al., 2010). To cope with the serious threat that misfolded proteins pose to the protein homeostasis network of eukaryotic cells, cells have evolved several protein quality control (PQC) strategies, including chaperone-assisted folding, proteasome degradation, autophagy-mediated protein turnover, etc. (Xu et al., 2018). And several of these pathways that have been shown to be related to CSPα will be described below.

### MAPS

Unconventional protein secretion refers to a collection of protein transport mechanisms that either export proteins lacking endoplasmic reticulum (ER) targeting signal sequences or transport proteins from the ER to the cell surface independent of the Golgi system (Xu et al., 2018). Misfolding-associated protein secretion (MAPS) is an unconventional protein processing mechanism specializing in exporting misfolded cytosolic proteins, including pathogenic proteins of various neurodegenerative diseases (Xu et al., 2018; Ye, 2018). USP19 is one of the substrate recognition molecules enriched on the surface of ER (Lee et al., 2018), and it has been found to be a regulator of MAPS (Xu et al., 2018). A previous study has defined USP19 as an ER-associated deubiquitinase (DUB) with chaperone activity and a C-terminal transmembrane domain. It binds to two major heat shock proteins in cells, HSC70 and HSP90, playing a role in PQC (Lee et al., 2016). It has been proven that overexpression of UPS19 may promote the release of some cytoplasmic proteins, while inactivation of UPS19 inhibits the secretion of unconventional proteins in mammalian cells (Lee et al., 2022a). Thus, as a membrane receptor, USP19 initiates the MAPS process when it recruits misfolded proteins to the ER surface. Subsequently, cargo proteins enter the lumen of late endosomes, which are usually located close to the ER. Then secretion occurs when a partial or complete membrane fuses between endosomes and the plasma membrane (Ye, 2018). Notably, the substrate specificity of MAPS is partly attributed to the intrinsic chaperone activity of USP19, with only ectopically expressed unassembled (and therefore misfolded) cytosolic proteins subject to USP19-mediated secretion, whereas correctly assembled endogenous counterparts are unaffected (Ye, 2018). Given the above, the elimination of misfolded proteins by MAPS appears to play a role in protein homeostasis maintenance.

How do cells secrete misfolded cytosolic proteins lacking a signal sequence? Multiple lines of evidence suggest that MAPS substrates may use one or more vesicle vectors as intermediate secretion compartments. Several types of vesicles, especially endolysosomes, are considered functional in unconventional secretion (Lee and Ye, 2018). The Golgi membrane lumen, known as a compartment for unconventional protein secretion (CUPS) in yeast, has been reported as a major mediator of nitrogen starvation-induced unconventional protein secretion (Malhotra, 2013). Protein trafficking to CUPS is thought to be mediated by the endosomal sorting complex required for transport (ESCRT) mechanisms and some autophagy regulators (Lee et al., 2022b). A recent study in mammalian cells has shown that the perinuclear membrane compartment adjacent to the Golgi system can serve as a CUPS equivalent compartment (Lee et al., 2022a).

In contrast, unconventional secretion of misfolded proteins in mammalian cells is ESCRT-independent and cannot be blocked by the autophagy inhibitor 3-MA (Lee et al., 2022b). Transfer of cargo into the lumen of CUPS appears to be mediated by a membrane protein named TMED10 (Zhang et al., 2020), a member of the EMP24/GP25L/P24 cargo receptor family (Strating and Martens, 2009), which is a single transmembrane protein localized to the ERGIC (ER and Golgi mid-septal compartment) and is involved in ER to Golgi trafficking. TMED10 may mediate transport by binding to consensus motifs in the cargo, thereby facilitating the transportation of cargo into the secretory compartment (Zhang et al., 2020).

**Figure 2:**
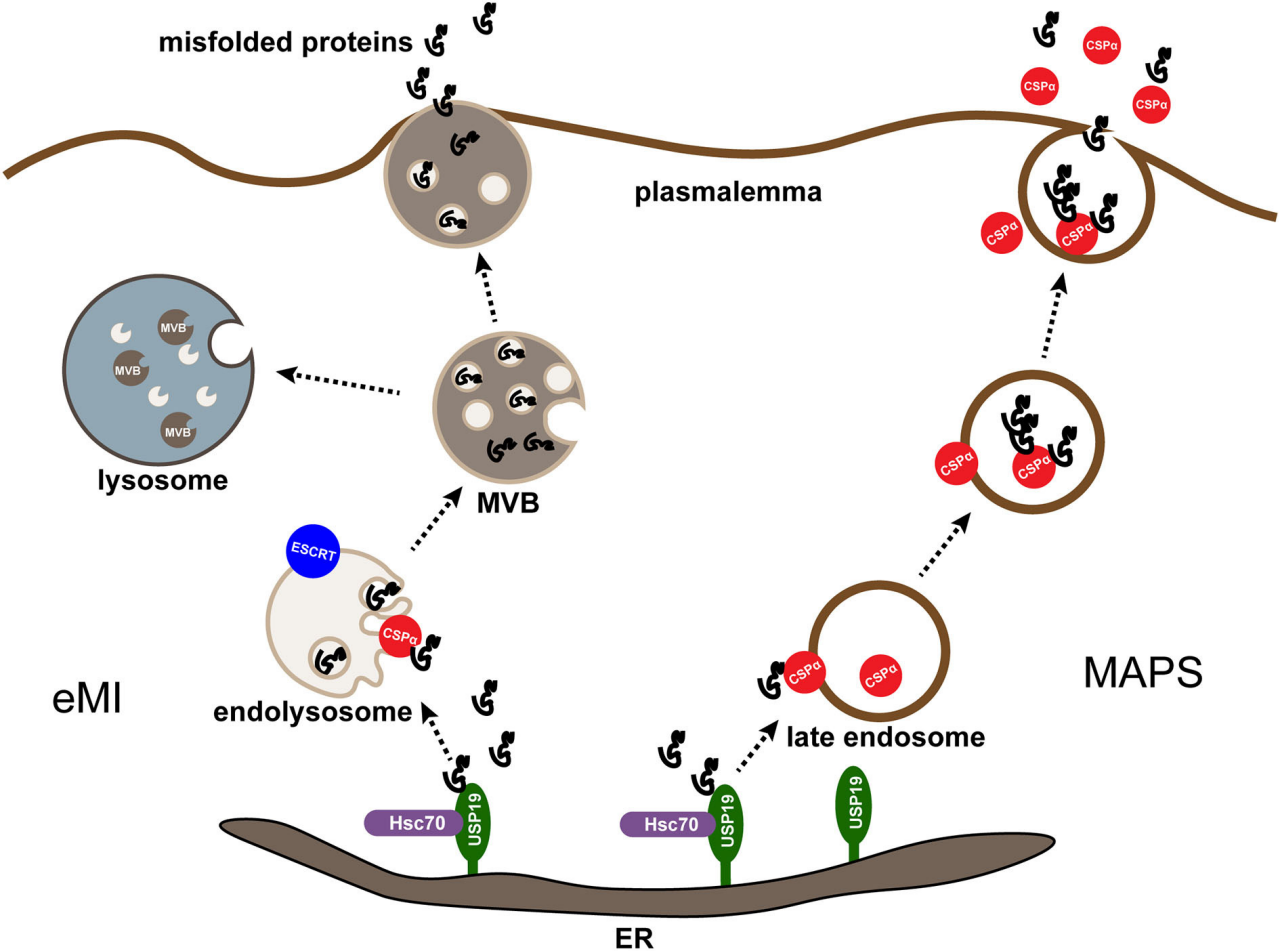
MAPS and eMI are two parallel pathways mediated by CSPα. Misfolding-associated protein secretion (MAPS) and endosomal microautophagy (eMI) are two protein quality control (PQC) mechanisms mediated by CSPα in mammalian cells. In both cases, an ER-associated deubiquitinase USP19 recruits misfolded substrates at the ER. For MAPS, misfolded proteins enriched in the ER by interacting with USP19 are transported to CSPα on the late endosome and taken up by endocytosis, subsequently they are transported to the plasmalemma for secretion and degraded in lysosomes from another cell. For eMI, CSPα escorts misfolded proteins into endolysosomes by an ESCRT-dependent mechanism. The resulting multivesicular bodies containing misfolded cargos can be degraded or secreted after the fusion of multivesicular bodies (MVBs) with lysosomes or plasma membrane.

CSPα has been reported to be involved in the unconventional secretion of misfolded cytosolic proteins through the MAPS process (Xu et al., 2018; Lee et al., 2022b). As discussed before, CSPα is associated with secretory granules in vesicle membrane neurons, synaptic vesicles, and endocrine neurosecretory and exocytotic cells (Huber, 2016). While during CSPα-stimulated secretion, cargo can be detected entering the late endosomal or pre-lysosomal compartment, like UPS19-dependent secretion. Besides, late endosome-associated CSPα shows extensive colocalization with MAPS substrates. These findings demonstrate that USP19-regulated MAPS and CSPα-dependent secretion may occur through a common membrane compartment (Ye, 2018). To further explore the relationship between CSPα and USP19, Xu, Y., and collaborators found that FLAG-tagged USP19 co-precipitated with a small portion of ectopically expressed CSPα but did not find endogenous CSPα and USP19 complex, indicating that USP19 did not form a stable interaction with CSPα (Xu et al., 2018). Both CSPα and HSC70 function downstream of USP19 because the downregulation of CSPα or HSC70 inhibits USP19-induced protein secretion (Xu et al., 2018). The misfolded proteins are initially enriched in the ER by interacting with USP19. Next, they are transported to CSPα in the late endosome and taken up by endocytosis. Subsequently, they are transported to the cell membrane for secretion and ultimately degraded in lysosomes from another cell (Ye, 2018) (Figure 2). Notably, neither USP19 nor CSPα is essential for MAPS, as knockdown of either of these genes results in only partial defects in MAPS, suggesting that functional redundancy with other membrane-associated chaperones or the existence of parallel secretion mechanisms (Lee et al., 2022a).

It is unclear to what extent MAPS contributes to the widely reported cell-to-cell transmission of misfolded proteins in neurodegenerative diseases (Xu et al., 2018). Studies on the relationship between the secretion of misfolded proteins and MAPS in neurodegenerative diseases have been frequently reported. For example, recent research has found that both cell lines and primary neurons can secrete ectopic expression and endogenous Tau through MAPS, raising the possibility that MAPS, if not combined with endocytosis-mediated clearance, may promote the formation of Tau-containing fibrils in the aging brain (Fontaine et al., 2016). Remarkably, although the mechanism of Aβ secretion is not fully understood, a recently published work has reported that it may also be exported through unconventional protein secretion channels (Nilsson et al., 2013). Additionally, in tissue culture cells, the secretion of α-synuclein, a major component of the pathological Lewy body of PD, is mainly mediated by MAPS (Ye, 2018). In contrast, ANCL disease caused by CSPα mutations is characterized by a large deposition of lipofuscin substance, most likely because the mutation affects the localization of CSPα to cysteine-rich fragments required for late endosomes and lysosomes. Thus, the material deposited in lysosomes may be the residue of MAPS vesicles accumulated due to secretion defects (Ye, 2018).

Interestingly, when MAPS is compared with chaperone-mediated autophagy (CMA), another PQC pathway, it is not uncommon to find that both pathways involve chaperones targeting soluble proteins on the membrane so that they can enter the membrane-enclosed lumen. At this point, though, the entry of misfolded proteins in MAPS into late endosomes is somewhat similar to that in CMA. There are striking distinctions and clear parallels between those two processes. For instance, the mechanisms by which proteins move across the membrane differ (Lee et al., 2018). Firstly, MAPS substrates are first recruited to the ER, while the substrates in CMA are directly delivered to lysosomes *via* HSC70. Secondly, MAPS substrates are recognized by USP19 because of their exposed hydrophobic segment, whereas CMA substrates use specific pentapeptide motifs for lysosomal targeting. Third, for CMA, substrates must enter lysosomes in an unfolded form. In contrast, in MAPS, EGFP or MCherry-labeled substrates are frequently detected in late endosomes by fluorescence microscopy, indicating that at least some of them enter late endosomes in folded form (Lee et al., 2018; Ye, 2018).

Furthermore, serum starvation enhances CAM-mediated degradation but does not enhance MAPS. One possible explanation is that serum starvation induces perinuclear localization of late endosomes/lysosomes, hindering their trafficking to the cell periphery and subsequent fusion with the plasma membrane (Lee et al., 2018). Finally, although CSPα localizes to late endosomes and lysosomes, its colocalization with MAPS substrates is only observed in late endosomes but not in lysosomes (Xu et al., 2018). For these reasons, it is rational to assume that MAPS and CMA use two different pathways to transport proteins to late endosomes or lysosomes (Ye, 2018). However, the similarity between the two approaches cannot be ignored. For instance, lysosomal-associated membrane protein-2 (LAMP2) is required in both pathways. LAMP2 is a glycoprotein that coats the luminal surface of late endosomes/lysosomes. LAMP2a, the specific isoform of LAMP2, has a unique C-terminal tail required for interaction with HSC70 and is intended to form a protein-conducting channel that allows entry of CMA substrates into the lumen of lysosomes (Kaushik and Cuervo, 2012). The knockdown of LAMP2 was found to attenuate MAPS, which, unlike CAM, could not be attributed to the channel function of the LAMP2a isoform because the secretion defect associated with LAMP2 knockdown could be fully rescued by overexpression of *DNAJC5*. An explanation for this is that the loss of LAMP2 may somehow interfere with the recruitment of *DNAJC5* to the substrates. Taken together, we can conclude that in MAPS, cargo uses a CAM-independent mechanism to enter the non-degradative pre-lysosomal compartment (Lee et al., 2018). Unfortunately, it is not known which LAMP2 isoform is involved in MAPS since LAMP2 isoforms are so nearly identical in amino acid sequence and molecular weight that antibodies cannot distinguish variations between them, and the similarity of the coding genes makes it difficult for siRNA to target specific isoforms (Lee et al., 2018).

Could MAPS help reduce the load of misfolded proteins, thereby promoting protein homeostasis? Quantitative immunoblot analysis showed that although MAPS could export a wide range of misfolded proteins, its capacity was limited. For any given substrate test, only a small fraction (5–10%) is ultimately secreted (Ye, 2018). On this basis, one potential interpretation is that both USP19 and CSPα contain an autoinhibitory domain, which limits the activity of USP19 and CSPα in this process. The autoinhibitory domain of USP19 is a ubiquitin-like-containing domain inserted between the ubiquitin-specific protease domains (Xu et al., 2018), and the autoinhibitory domain of CSPα is the HSC70-bound J-domain (Lee et al., 2022b). When these autoinhibitory domains are removed, the resulting truncated protein is significantly more active than its WT counterpart in MAPS (Lee et al., 2022a). In addition, many MAPS substrates have also been reported to be degraded by the 26S proteasome (Ye, 2018; Lee et al., 2022a), and UPS19-deficient cells are more sensitive to proteasome inhibitor-induced cytotoxicity (Lee et al., 2016), suggesting that MAPS may only serve as a complementary PQC mechanism.

### Endosomal microautophagy

Endosomal microautophagy (eMI) is a special form of autophagy. In the process, the late endosomes ingest cytoplasmic materials through membrane invagination and pinching to form multivesicular bodies (MVBs, also known as caveolae). Subsequently, MVBs degrade intracellular substances (Oku and Sakai, 2018). Earlier studies considered eMI largely non-selective, but *in vitro* remodeling systems demonstrated that cytosolic proteins containing the KFERQ motif can be directly translocated to late endosomes depending on HSC70, KFERQ, and ESCRT (Morozova et al., 2016), confirming the existence of selective eMI. In addition to KFERQ-dependent selective eMI, studies have shown that CSPα is also involved in selective eMI (Lee et al., 2022b). Despite lacking a KFERQ motif, endolysosome-associated CSPα can efficiently enter multivesicular bodies with its conjugate (Lee and Ye, 2018). This process involves an ESCRT mechanism but is independent of the J-domain of CSPα (Lee et al., 2022b). Moreover, according to the latest research, lipofuscin accumulation in ANCL associated with CSPα mutations may be caused by abnormal membrane flow due to an imbalance between unconventional protein secretion and eMI (Lee et al., 2022a). Taking these aspects into consideration, although how CSPα recruits substrates to endolysosomes and cooperators with HSC70 in eMI remains to be determined, the role of CSPα in eMI and unconventional protein secretion significantly expands the functional repertoire of CSPα, which makes it a critical regulator of protein quality control (Lee et al., 2022b).

What calls for special attention is that CSPα activation stimulates both MAPS and eMI. Therefore, combining these two PQC pathways may help prevent misfolded proteins and membrane overflow into endolysosomes, thereby inhibiting lipofuscin biogenesis (Lee et al., 2022a). In effect, MAPS and eMI pathways have long been considered tightly coupled, as conditions that increase eMI also stimulate MAPS (Lee et al., 2022a). However, Lee and collaborators have parallel proved the relationship between those two PQC pathways. More correctly, they are two parallel but functionally coupled quality control processes mediated by CSPα (Lee et al., 2022b) (Figure 2). Lee and collaborators' study where immunoblotting analysis of conditioned media and cell lysates from cells transfected with MAPS model substrate GFP1-10 showed partial detection of GFP1-10 in the media, and co-expression of *DNAJC5* enhanced GFP1-10 secretion (Lee et al., 2022a), consistent with previous reports (Fontaine et al., 2016; Xu et al., 2018). Yet, when GFP1-10 secretion was examined in the presence of the VPS4 dominant negative (DN) mutant 228q, which may block ESCRT-mediated eIM, it was surprising that MAPS substrate GFP1-10 secretion was significantly increased under both basal and *DNAJC5* overexpression conditions (Lee et al., 2022b). Besides, the J domain-deleted CSPα mutant, which has been proven to promote MAPS, has a significantly increased activity in promoting α-synuclein secretion, it only modestly promotes the translocation of α-synuclein into endolysosomes (Lee et al., 2022b). Also of note is that ANCL-associated *DNAJC5* mutations (both p.L115R and p.L116δ) abolish CSPα function in MAPS but maintain its eIM-stimulating activity (Lee et al., 2022b), demonstrating that CSPα palmitoylation is essential for MAPS but dispensable for *DNAJC5-*mediated eMI. Given all that, MAPS and eMI are apparently two parallel pathways.

### Ubiquitin proteasome system

In age-related neurological dysfunction, misfolded neurotoxic proteins are typically ubiquitinated and then targeted for degradation, which is thought to be a protective mechanism as brain cells try to cope with neurodegeneration (Johnson et al., 2010). Disruption of clearance of protein aggregates *via* the ubiquitin-proteasome (UPS) pathway has also been found to play a role in the pathogenesis of several neurodegenerative diseases, such as late-onset PD and Huntington's disease (Saba et al., 2008). Then, is there any relevance between UPS and CSPα as different mechanisms for eliminating misfolded proteins? As mentioned in the previous section, the UPS can also degrade various MAPS substrates, suggesting that MAPS and the UPS represent two alternative pathways for handling misfolded or unwanted proteins. UPS has been proven more effective at eliminating misfolded proteins, yet the MAPS pathway contributes to protein homeostasis because USP19-deficient cells are highly sensitive to proteasome inhibitor treatment (Lee et al., 2014). As a matter of fact, the UPS pathway, like MAPS, is thought to be related to CSPα (McCue et al., 2015). McCue and collaborators explored the mechanism of ANCL disease progression by using *Dnj-*14 mutant strains of homologous nematodes of *DNAJC5*. The transcriptional analysis of these mutants revealed extensive down-regulation of genes involved in UPS compared with control strains, especially the polycyclic E3 ubiquitin ligase components, including F-box proteins, SKp1-related (SKR) proteins, and BTB proteins in this strain, and significantly, degradation of ubiquitinated proteins is reduced in mutant worm strains (McCue et al., 2015). The study demonstrates that disruption of the important synaptic chaperone CSPα results in changes in the expression levels of UPS-associated proteins and has a knock-on effect on overall protein degradation in Caenorhabditis elegans (McCue et al., 2015).

Contrary to this view, Sharma and collaborators found that in CSP KO mice, the expression of proteins involved in proteasome degradation was increased, proteasome activity was extensively enhanced, and the ubiquitination levels of SNAP-25 were increased by about 40% (Sharma et al., 2012a). However, this observation was based on immunoblotting and fluorometric assays of proteasome active site activity for a select few proteasome proteins but not components of polyE3 ubiquitin ligase. Therefore, it seems that more evidence is needed to clarify whether CSPα enhances or inhibits the function of the UPS system. In addition, based on the type of disease or system, reports vary as to whether direct inhibition of the proteasome is harmful or beneficial (McCue et al., 2015). For example, inhibition of proteasome activity has been shown to be advantageous in CSPα-deficient mouse models in protecting against β-amyloid neurotoxicity (Favit et al., 2000). However, proteasome inhibitor treatment itself may cause neurodegeneration (Romero-Granados et al., 2011). Overall, several ubiquitin ligases have been identified as promising drug targets. In particular, progress has been made in developing specific inhibitors of SCF complexes (SKPL-Cull-F-box protein, a member of the ubiquitin ligase family) (McCue et al., 2015).

## Conclusion

ANCL, a form of NCL, is an inherited neurodegenerative disease with progressive neuronal dysfunction characterized by neuronal death and lipofuscin deposition in the neuronal or non-neuronal lysosomes. Although mutations in CSPα, a protein-encoding 35kDa in the human *DNAJC5* gene, are known to be associated with ANCL, the pathogenic mechanisms involved remain unknown. The most well-studied function of CSPα is its cytoplasmic chaperone function. CSPα is abundant in presynaptic vesicles, interacting with HSC70 to ensure correct protein folding. Mutant CSPα causes loss of palmitoylation, mislocalization, and aggregation of CSPα, which then triggers a series of reactions and destabilizes key proteins related to its function, such as synaptic SNAP-25 proteins and PPT1 proteins. Although the exact mechanism is unknown, certain changes in these proteins contribute to NCL.

In numerous animal and human studies, defects in CSPα have been shown to cause neurodegeneration. In addition to ANCL, AD, PD, FTD, and Huntington have been shown to be associated with CSPα. Although the mechanisms of these neurodegenerative diseases have not been fully explained, neurodegenerative diseases are often associated with protein misfolding. Further studies have revealed that CSPα is essential for transporting misfolded proteins. CSPα has been shown to be associated with lysosomal degradation. Inadequate lysosomal degradation can lead to abnormal membrane flow and misfolded protein entry into endolysosomes, thus leading to error-prone protein accumulation. Additionally, CSPα has been shown to be involved in MAPS, eMI, and UPS processes, which are known to play important roles in maintaining the stability of misfolded proteins. However, all the relevant mechanisms researches are not detailed enough, and more evidence is required to reveal the deeper molecular mechanisms.

## Author contributions

LH edited the draft. ZZ reviewed, edited and proofed the manuscript. All authors contributed to the article and approved the submitted version.

## Funding

This review was supported by the Nature Science Foundation of China (Grant No. 82071183) to ZZ.

## Conflict of interest

The authors declare that the research was conducted in the absence of any commercial or financial relationships that could be construed as a potential conflict of interest.

## Publisher's note

All claims expressed in this article are solely those of the authors and do not necessarily represent those of their affiliated organizations, or those of the publisher, the editors and the reviewers. Any product that may be evaluated in this article, or claim that may be made by its manufacturer, is not guaranteed or endorsed by the publisher.
